# Migratory pattern of the coronavirus disease 2019 and high fatality rates among kidney transplant recipients: report from the Brazilian Multicenter Cohort Study

**DOI:** 10.1590/2175-8239-JBN-2021-0063

**Published:** 2021-07-28

**Authors:** Marina Pontello Cristelli, Tainá Veras de Sandes-Freitas, Laila Almeida Viana, Lúcio R. Requião-Moura, Luis Gustavo Modelli de Andrade, Helio Tedesco-Silva, José Medina-Pestana

**Affiliations:** 1 Universidade Federal de São Paulo, Hospital do Rim, São Paulo, SP, Brasil.; 2 Universidade Federal do Ceara, Departamento de Medicina Clínica, Fortaleza, CE, Brasil.; 3 Universidade Estadual Paulista Júlio de Mesquita Filho, Faculdade de Medicina, Botucatu, SP, Brasil.; 4 Hospital Geral de Fortaleza, Papicu, Fortaleza, CE, Brasil.; 5 Hospital Israelita Albert Einstein, Transplant Division, São Paulo, SP, Brasil.

**Keywords:** Kidney Transplant, Coronavirus, Mortality, Transplante Renal, Coronavírus, Mortalidade

## Abstract

**Introduction:**

The unprecedented coronavirus disease 2019 (COVID-19) pandemic has affected kidney transplant (KT) recipients, with worldwide fatality rates around 25%. Considering the well-known Brazilian socio-demographic disparities, this report describes for the first time the main outcomes of COVID-19 in KT recipients according to Brazilian geographic regions.

**Methods:**

This multicenter national retrospective analysis included data from KT recipients with confirmed COVID-19 between March and November 2020.

**Results:**

Thirty-five of the 81 centers (57% of KT activity in Brazil) reported 1,680 patients with COVID-19. The Northeast was the first to reach the peak in the number of infections. The Southeast, due to its population density, contributed with the largest number of patients. Patients had a median age of 52 years, 76% had hypertension and 34% diabetes, 75% were recipients of a deceased donor, and the time interval between diagnosis and transplantation was 5.9 years. In 53% of patients, immunosuppression was adjusted, and clinical support varied according to geographic region. Hospitalization was required for 65% of the patients, 35% of them needed intensive care, 25% mechanical ventilation, and 23% renal replacement therapy. The 90-day overall fatality was 21%, being 23% in the Southeast, 16% in the Northeast, and 19% in the Central-west and South regions.

**Conclusion:**

The migratory pattern of the pandemic among KT recipients followed that of the general population and the outcomes were influenced by regional features. COVID-19 in KT recipients was associated with high utilization of health-care resources and higher fatality rates than those reported in the general population.

## Resumo

**Introdução:**

A pandemia da COVID-19 afetou receptores de transplante renal (TR), com taxas de mortalidade mundial em torno de 25%. Considerando as notórias disparidades sócio-demográficas brasileiras, este relatório descreve pela primeira vez principais características e desfechos da doença em receptores de TR, segundo as regiões geográficas.

**Métodos:**

Esta análise retrospectiva multicêntrica nacional incluiu dados de receptores de TR com COVID-19 confirmada entre Março/Novembro de 2020.

**Resultados:**

Trinta e cinco dos 81 centros (57% da atividade de transplante renal brasileira) relataram 1.680 pacientes com COVID-19. O Nordeste foi o primeiro a atingir o pico no número de infecções. O Sudeste, por sua densidade populacional, contribuiu com maior número de pacientes. Pacientes tinham em média 52 anos, 76% apresentavam hipertensão e 34% diabetes, 75% receptores de doador falecido e o tempo entre diagnóstico e transplante foi de 5,9 anos. Em 53% dos pacientes, os imunossupressores foram ajustados, e o tratamento variou segundo a região. Hospitalização foi necessária para 65% dos pacientes, 35% necessitaram de cuidados intensivos, 25% ventilação mecânica, e 23% terapia renal substitutiva. A mortalidade geral em 90 dias foi 21%, sendo 23% no Sudeste, 16% no Nordeste, e 19% nas regiões Centro-Oeste e Sul.

**Conclusão:**

O padrão migratório da pandemia entre os receptores de TR seguiu o da população em geral e os desfechos foram influenciados por características regionais. A COVID-19 em receptores de TR foi associada à alta utilização de recursos de saúde e taxas de mortalidade mais altas do que as relatadas na população em geral.

## Introduction

Less than one year after confirmation of the first SARS-CoV-2 infection in China, coronavirus disease 2019 (COVID-19) evolved into an unprecedented pandemic. In Brazil, since February 2021, the number of confirmed infections has overcome 8.5 million people, resulting in 200 thousand deaths, recorded in all 27 states of the country^1^.

As observed with most infectious diseases, patients with chronic kidney disease (CKD) have shown a higher risk of worse COVID-19-associated clinical outcomes, further heightened by increasing age and the presence of comorbidities such as diabetes and cardiovascular disease. Kidney transplant recipients, presenting various stages of CKD and comorbidities in addition to chronic use of immunosuppressive drugs, have shown even higher fatality rates, at around 25% worldwide^2^^-^ 5.

Brazil has one of the leading transplantation programs, reaching the second largest program in terms of absolute number of kidney transplants in the world^6^. Anticipating the significant negative impact of the SARS-CoV-2 infection in patient management and outcome, we established a multicenter national registry database on 21^th^ May 2020, aiming to analyze the main features of this disease among KT recipients. Considering the described associations between COVID-19 and demographic, socioeconomic, and access to health services' disparities^7^, this first report seeks to describe relevant data stratified by the five regions of our country.

## Methods

This is a national multicenter cohort study. All 81 active Brazilian transplant centers were invited to participate and, at the time of this first analysis, 35 centers had effectively completed the regulatory processes and enrolled patients, which represents 57% of the all national transplantation activity. Kidney transplant recipients of any age, who were transplanted at any time, and who were diagnosed with COVID-19 between March and November, 2020 were eligible for this study. The diagnosis was established when patients presented at least one COVID-19-attributed symptom or sign, followed by a positive reverse-transcription polymerase (RT-PCR) assay, serologic tests, or antigen detection. Screening diagnoses in asymptomatic patients were excluded. A dedicated web-based clinical research form was developed to upload individual anonymized data captured for each patient in each transplant center. Key and discordant data were adjudicated. The study was approved by the National Ethics Research Committee (CAEE 30631820.0.1001.8098) and by the local ethics committee of all participating centers. Informed consent was obtained from all patients. The fatality rate was calculated by the proportion of deaths due to COVID-19 in the total number of patients diagnosed and it was grouped according to intermediate outcomes: hospitalization, acute kidney injury (AKI) and intensive care unit (UCI), mechanical ventilation (VM), and renal replacement therapy (RRT) requirement. Patients were stratified by regions of the country where the centers are located. The final follow-up date for the present analysis was December 11^(th)^, 2020 or the date of death.

## Results

**Figure 1:**
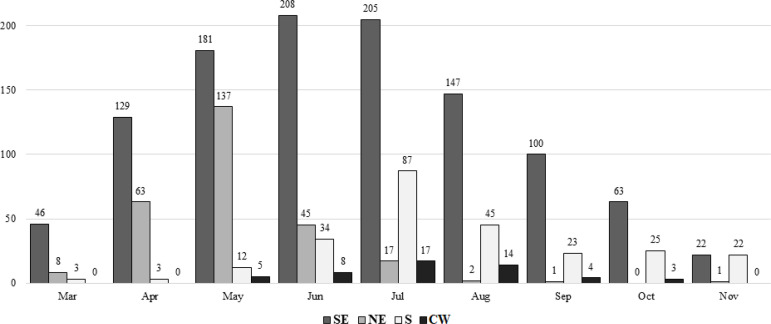
Monthly number of kidney transplant patients with COVID-19, stratified by region.

From the 3^rd^ of March to the 30^th^ of November 2020, 26 of the 1706 eligible patients were excluded for being diagnosed but asymptomatic, resulting in 1680 patients with confirmed COVID-19 effectively included. The temporal distribution of patients according to geographic location is shown in Figure 1. The incidence of COVID-19 among KT recipients followed that of the regional population, being higher in the Southwest and South regions. The increase in the number of infections started primarily in the Northeast region, being the first to peak in relation to its first case. Progressively, the Southeast, Central-west, and South regions reached their peaks during the following months. A similar pattern of recovery was observed, with subsequent reduction in the number of infections after implementation of the non-pharmacological measures such as mobility restrictions, social distancing, use of masks, and hand hygiene.

Table 1 summarizes the baseline characteristics of the patients. They were mostly middle-aged male recipients from deceased donors, with a prevalence of diabetes of 34% and obesity of 24%, with a long vintage of transplantation. In the Northeast, the first region to be affected, patients were younger, fewer were obese, and presented with better baseline kidney graft function. Despite the predominance of long-term transplantation, COVID-19 was diagnosed among recipients in the first 30 days after transplantation in the South and Southeast regions.

**Table 1:** Baseline characteristics and clinical management of the kidney transplant recipients with COVID-19, stratified by Brazilian regions where the transplant center is located

Variable	Overall N=1,680	Central-west N=51	Northeast N=274	South N=254	Southeast N=1,101
**Baseline characteristics**					
Age, median (IQR)	52 (42-60)	49 (39-58)	50 (40-59)	52 (40-58)	53 (43-61)
Sex (male) - n (%)	1.015 (60.4)	27 (52.9)	158 (57.7)	160 (63.0)	670 (60.9)
Ethnicity (African-Brazilian) - n (%)	191 (11.3)	6 (11.8)	27 (9.9)	21 (8.3)	137 (12.4)
Comorbidities					
Hypertension - n (%)	1.272 (75.7)	44 (86.3)	217 (79.2)	203 (79.9)	808 (73.4)
Diabetes - n (%)	571 (33.9)	17 (33.3)	105 (38.3)	90 (35.4)	359 (32.6)
Obesity - n (%)*	378 (23.8)	7 (13.7)	47 (18.1)	74 (30.0)	250 (24.3)
Deceased donor - n (%)	1.256 (74.7)	36 (70.6)	212 (77.4)	304 (80.3)	704 (63.9)
Baseline eGFR, median (IQR)	48.4 (32.4-65.6)	49.6 (32.5-68.7)	56.5 (39.9-73.6)	45.9 (32.0- 65.4)	46.5 (31.4-63.2)
**COVID-19 infection**					
Time from transplant to diagnosis, months - median (IQR)	5.9 (2.3-10.7)	8.5 (4.0; 13.2)	6.7 (2.8; 11.9)	4.7 (1.2; 9.7)	6.0 (2.3; 10.5)
*First month after transplantation* *- n (%)*	41 (2.4%)	0 (0.0)	4 (1.5)	15 (5.9)	22 (2.0)
Community source of infection - n (%)^#^	1.526 (91.7)	49 (98.0)	254 (93.0)	205 (84.4)	1.018 (92.7)
**Clinical management**					
Azithromycin - n (%)	811 (48.2)	15 (29.4)	192 (70.1)	112 (44.1)	492 (44.7)
Hydroxychloroquine or chloroquine - n (%)	192 (11.4)	3 (5.9)	70 (25.5)	15 (5.5)	104 (9.4)
High dose of steroids - n (%)	550 (32.7)	23 (45.1)	124 (43.1)	94 (37.0)	309 (28.1)
Adjustment in immunosuppression - n (%)	908 (54.0)	27 (52.9)	155 (56.6)	143 (56.3)	583 (53.0)
*Immunosuppression discontinuation - n (%)*	399 (23.8)	11 (21.6)	41 (15.0)	33 (13.0)	314 (28.5)

IQR: interquartile range; eGFR: estimated creatinine glomerular filtration rate by CKD EPI equation in mL/min/1.73 m^2^

* missing-value: 92;

# missing-value: 16.

Regarding the management of COVID-19 and the immunosuppressive therapy (Table 1), the most frequent interventions were azithromycin prescription and use of high-dose steroids. The Northeast led the use of antibiotics and chloroquine (or hydroxychloroquine), whereas the Central-west and the South had the lowest rates of utilization. Most patients did not discontinue immunosuppression; however, at least 53% of patients in all regions required some type of dose adjustments or discontinuations, primarily of the antiproliferative drugs. Reduction of the immunosuppressive therapy was less frequent in the Northeast and South regions.

Of the 1,680 patients, 65% required hospitalization, 35% of the hospitalized patients required ICU, 25% developed severe acute respiratory syndrome requiring MV, and 23% needed RRT. These results are detailed in Table 2. The Northeast was the region with the lowest rates of hospitalization, as well as ICU and RRT requirement. Although the South region had a lower incidence of AKI, the need for RRT was similar to that observed in other regions. The 90-day overall fatality rate after COVID-19 diagnosis was 21%, whereas this rate was 32% among patients who required hospitalization, 60% among those who required ICU admission, and 78% among those who needed MV. When stratified by region, the fatality rate was 23% in Southeast, followed by 19% in Central-west and Southern regions, and 16% in Northeast.

**Table 2:** Major outcomes of the kidney transplant recipients with COVID-19, stratified by Brazilian region

Outcomes, n (%)	Overalll N=1,680	Central-west N=51	Northeast N=274	South N=254	Southeast N=1,101
Hospitalization	1,094 (65.1)	33 (64.7)	143 (52.2)	163 (64.2)	755 (68.6)
Fatality	355 (32.4)	10 (30.3)	43 (30.1)	48 (29.4)	254 (33.6)
AKI at the COVID-19 diagnosis*	244 (23.2)	13 (29.5)	47 (21.9)	21 (15.7)	163 (24.8)
Fatality	87 (35.7)	5 (38.5)	18 (38.3)	7 (33.3)	57 (35.0)
ICU requirement^#^	577 (34.6)	17 (33.3)	68 (24.9)	78 (31.6)	414 (37.7)
Fatality	344 (59.6)	10 (58.8)	43 (63.2)	43 (55.1)	248 (59.9)
MV requirement^#^	415 (24.9)	11 (21.6)	52 (19.0)	55 (22.1)	297 (27.1)
Fatality	322 (77.6)	10 (90.9)	41 (78.8)	37 (67.3)	234 (78.8)
RRT requirement^#^	391 (23.4)	12 (23.5)	51 (18.7)	59 (23.6)	269 (24.5)
Fatality	280 (71.6)	10 (83.3)	37 (72.5)	35 (59.3)	198 (73.6)
Overall fatality	355 (21.1)	10 (19.6)	43 (15.7)	48 (18.9)	254 (23)

AKI, acute kidney injury; ICU, intensive care unit; MV, mechanical ventilation; RRT, renal replacement therapy.

* missing-value: 628;

# missing-value: 10.

## Discussion

The present report demonstrates how the pandemic affected the KT recipients across the different regions of Brazil. Similar to what happened in the general population, the first cases were reported in the Southeast region, which includes Sao Paulo, a megalopolis with more than 11 million inhabitants, which has had the highest number of COVID-19 patients over the pandemic to date^7^. After this first phase, when the spreading of SARS-CoV-2 infection occurred mainly from cities with large influx of people (Sao Paulo, Recife, Rio de Janeiro, for instance) and within state borders, the dissemination took place through long distance travelling and reached smaller and more remote cities in the country. Although the Northern region of Brazil is currently showing high numbers of SARS-CoV-2 infections^8^, there were no reported cases of COVID-19 in KT recipients until the database lock date for this analysis, because the centers in that region had not completed the regulatory process for this first report. Of note, the transplant activity in the North region corresponds only to 2% of the Brazilian transplant activity^6^, and patients with end stage chronic kidney disease are usually referred to transplantation in the other regions. In contrast, the Southeast region reported the highest number of cases over time, once this region contributes with 53% of the transplant activity in Brazil^6^.

As the pandemic peaked in the Northeast, the use of off-label therapies to manage the COVID-19 infection was more prominent in this region. The evolving knowledge, especially regarding the efficacy and safety of azithromycin and chloroquine/hydroxychloroquine^9^, was associated with the decrease of the use of these therapies in the other regions of the country. On the other hand, demographic characteristics of the patients in the Northeast, such as lower obesity rates, might have resulted in a less severe disease, evidenced by the lower hospitalization rates, ICU admission, immunosuppressive drug discontinuation, and ultimately lower fatality rate. However, because of the pandemic dynamics through the different regions, it is impossible to formally analyze the variables and outcomes and therefore make robust statements about these impressions.

KT patients with COVID-19 had an eight-fold higher hospitalization rate and a ten-fold higher fatality rate when compared to that observed in the general Brazilian population^7^^,^ 8 Although this subgroup of patients is almost 10 years younger than the reported hospitalized patients in the general population with COVID-19^7^, they usually present multiple comorbidities in addition to chronic renal function impairment and are under the unavoidable use of immunosuppression medication, which would justify the higher disease severity.

## Conclusion

COVID-19 among KT recipients followed the migratory characteristic of the pandemic in Brazil, affecting all regions over time. This cluster of patients is at higher risk for poor outcomes of COVID-19 and should receive special sanitary attention and be prioritized for vaccination.
